# Digital innovation through partnership between nature conservation organisations and academia: A qualitative impact assessment

**DOI:** 10.1007/s13280-015-0704-2

**Published:** 2015-10-27

**Authors:** Carlos Galán-Díaz, Peter Edwards, John D. Nelson, René van der Wal

**Affiliations:** dot.rural (RCUK Digital Economy Research), University of Aberdeen, MacRobert Building, King’s College, Aberdeen, AB24 5UA UK; Aberdeen Centre for Environmental Sustainability (ACES), School of Biological Sciences, University of Aberdeen, Auris, 23 St. Machar Drive, Aberdeen, AB24 3UU UK

**Keywords:** Digital technologies, Impact, Impact assessment, Nature conservation, Partnership working with academia

## Abstract

Nature conservation organisations increasingly turn to new digital technologies to help deliver conservation objectives. This has led to collaborative forms of working with academia to spearhead digital innovation. Through in-depth interviews with three UK research-council-funded case studies, we show that by working with academics conservation organisations can receive positive and negative impacts, some of which cut across their operations. Positive impacts include new ways of engaging with audiences, improved data workflows, financial benefits, capacity building and the necessary digital infrastructure to help them influence policy. Negative impacts include the time and resources required to learn new skills and sustain new technologies, managing different organisational objectives and shifts in working practices as a result of the new technologies. Most importantly, collaboration with academics was shown to bring the opportunity of a profound change in perspectives on technologies with benefits to the partner organisations and individuals therein.

## Introduction

The adoption of new digital technologies by nature conservation organisations, such as GPS enabled mobile devices, interlinked databases and high-performance computing, has led to state changes in a wide range of dimensions including data gathering, public engagement, increased knowledge and skills, and monitoring (e.g. Bonney et al. 2009; Dickinson et al. 2012; Miller-Rushing et al. 2012; Arts et al. 2015). Inevitably, the use of digital technologies has not only brought positive outcomes, but also created challenges in the ways people interact with nature, such as potential exclusion of certain groups that are not technologically-minded and the risk of volunteer fatigue in digitally enforced public engagement activities (e.g. Newman et al. 2012; Roy et al. 2012). In spite of this, nature conservation organisations adopt new digital technologies because of the belief that these may help them to deliver the plurality of conservation and organisational objectives more efficiently (see Verma et al. 2015).

For new digital technologies to be adopted, nature conservation organisations require technological expertise that is not typically found within their institutions (Arts et al. 2013). Partnering with academics is one of the ways through which this expertise shortage can be addressed. Such partnerships primarily concern the co-working of ecology and computing sciences (e.g. Jepson and Ladle 2015; Joppa 2015; Saito et al. 2015), and the number of such constellations is rapidly rising (Arts et al. 2015). Despite their increasing prevalence, the positive and negative impacts of collaborations between conservation organisations and academia remain poorly understood.

Over the last two decades, the global funding landscape has emphasised that publicly financed research ought to be receptive to the needs of users, national economies and wider society. Such a redrawing of the ‘contract between science and society’ (e.g. Gibbons 1999) incited forms of participation, collaboration and knowledge exchange between researchers and non-academic stakeholders, and led to the understanding that great gains can be made where such interactions are bi-directional rather than non-academic stakeholders being passive recipients of academic expertise (Abreu et al. 2009; O’Brien et al. 2013). The proliferation of such more participatory research motivated both funders and researchers to seek ways to account for the impacts of research outside academia (e.g. Nature special issue on Impact 2013).

Impact from research, both academic and non-academic, can be evaluated via quantitative and qualitative approaches. An example of a quantitative approach is STAR METRICS (Science and Technology in America’s Reinvestment—Measuring the EffecTs of Research on Innovation, Competitiveness and Science), an empirical infrastructure that, through the development of bottom-up, standard and auditable measures, “document(s) the outcomes of science investments to the public”. An example of a qualitative approach is part of the Research Excellence Framework (REF) developed by the Higher Education Funding Council in the UK, where a series of expert panels are used to assess ‘impact narratives’ (texts describing the impacts of particular research projects to provide corroboration of the impact claims) in terms of the research’s *reach* and *significance* of the impact (REF 2013). At its broadest, impact is defined by RCUK (2007) as “the demonstrable contribution that excellent research makes to society and the economy”. It is recognised that researchers cannot predict the impact of their research but that they can explore, from the outset, who could potentially benefit from their work in the longer term and how they may maximise the chances for this to happen (Payne-Gifford 2014).

What unites the rhetoric on measurement of non-academic impact of research (e.g. European Science Foundation 2012; Research Excellence Framework 2014; National Science Foundation 2014) is that they are success-oriented exercises or seek to account for the benefits of the research, and thus reduce the likelihood of accounting for less beneficial or detrimental aspects. In our work, we aimed to reveal the impact of partnership working with academia on nature conservation organisations and draw out both positive and negative dimensions. For this, we focused on three RCUK-funded projects in which teams of scientists (from the computing, ecological and social sciences) worked together with nature conservation organisations to achieve certain digital innovations.

## Materials and methods

### Case studies

Our investigation focused on three ‘digital innovation in nature conservation’ projects which were part of a large RCUK-funded interdisciplinary research centre (dot.rural). The first case study involved the Royal Society for the Protection of Birds (RSPB), which is concerned with the conservation of wild birds, other wildlife and the places in which they live in a wide variety of ways. dot.rural’s collaboration with the RSPB resulted in a dedicated web platform (Blogging Birds), which portrayed automatically generated blogs that captured the movements of satellite-tagged red kites (*Milvus milvus*), and through which the public could engage with the life of this (reintroduced) species (Ponnamperuma et al. 2013; Van der Wal et al. 2015b). The partnership working involved interactions (face-to-face meetings, additional email and phone conversations) between a core academic team (of three computing scientists and two ecologists) and an RSPB conservation officer, with additional (regional and national, including higher management) RSPB staff involved on a more ad-hoc basis (in-person meetings at their premises).

The second case study involved the Scottish Mink Initiative (SMI), a community-based endeavour aimed at protecting native wildlife and river fishing interests by removing the invasive non-native American mink (*Neovison vison*). Through the collaboration, an infrastructure for online data gathering and volunteer feedback provision in real-time was developed (Tintarev et al. 2012; Webster et al. 2014). The core academic team, consisting of four computing scientists, one social scientist and two ecologists, was in close contact with three SMI staff, one of which took the role of project liaison and attended as many of the weekly to monthly meetings as possible and fed back to other SMI staff. This individual (two different people occupied this role over the course of the project) was also in close email contact with some of the academic partners to help guide the development of the digital tool (MinkApp). Annual day-long meetings involving the much wider pool of SMI (and related) staff (of variable composition) were also held to discuss problems, progress and ways forward.

**Fig. 1:**
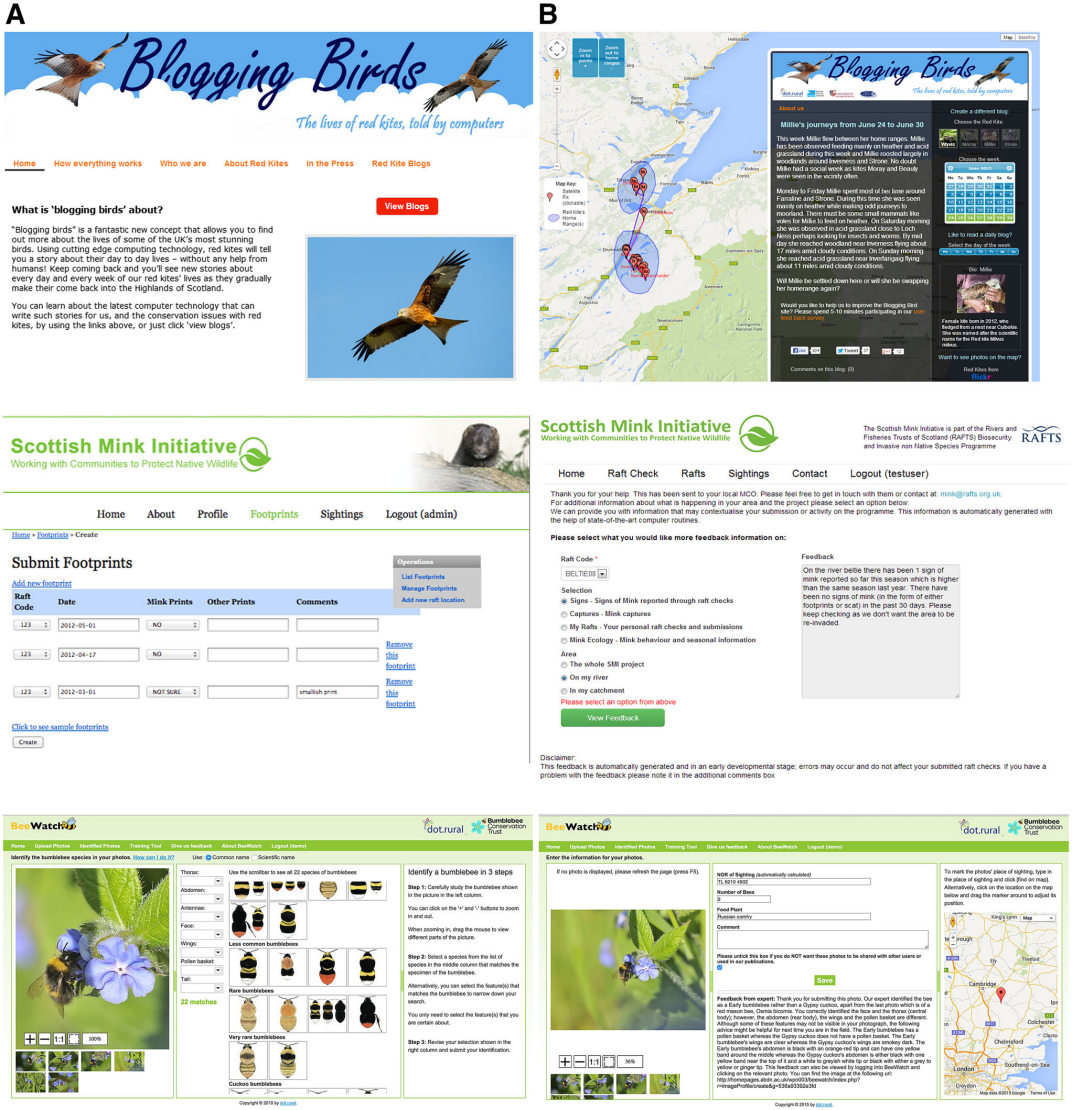
Screenshots of the different platforms developed with each of the organisations. *Top panel* RSPB—Blogging Birds entry page (**a**) and red kite journey map and blog (**b**). Upon selecting a specific kite and week, available location data are shown as geo-tags on the Google Map together with an automatically generated blog describing the kites’ journey. *Middle panel* SMI—mink data submission page (**a**) and feedback page (**b**). After having submitted presence or absence of mink signs on their raft, a user can request feedback about different aspects of mink and their management, and at different geographical scales. This feedback is automatically generated and aimed to contextualise the observation just submitted. *Bottom panel* BBCT—BeeWatch species identification page (**a**) and feedback page (**b**). After uploading a photo of a bumblebee, a digital key can be used to work out the identity of the specimen. Upon submitting the identification, automatically generated feedback is given to the user

The third case study, involved the Bumblebee Conservation Trust (BBCT), an organisation supporting wildlife and habitat diversity to halt the decline of bumblebees across the UK. In this collaboration an online submission portal with identification tool and consensus identification functionality was developed (BeeWatch); uniquely, submitters receive real-time feedback so that contributors can improve their ID skills (Blake et al. 2012; Van der Wal et al. 2015a). Partnership working varied in intensity over the course of the project and seasonally. Overall plans for tool development were worked out during annual face-to-face meetings between the academic team (five computing scientists and three ecologists) and BBCT (four) staff. During the first two years of the project there was intense collaboration (over bumblebee identifications and press-related activities) during summer (between one of the University’s ecologists and two BBCT staff); during the remainder of the time interaction was more ad-hoc over the phone and by email. Figure 1 shows screenshots of each of the technologies co-developed with the various organisations.

### Initial methodological framework

We reviewed different large-scale initiatives that explicitly consider the evaluation of research impacts outside academia (Swiss National Science Foundation 2007; RCUK Pathways to Impact 2010; European Science Foundation 2012; Research Excellence Framework 2014; National Science Foundation 2014). We identified five impact types that cut across these initiatives, and used them as the basis of an initial framework (Table 1) which describes the different impact types and provides examples for each of these.

**Table 1:** Impact types and examples of indicators used to evaluate collaborations between nature conservation organisations and academia

Type of impact	Indicators or changes/influences on
Social	Public engagement Cultural enrichment Quality of life enrichment
Professional practice	Innovation in products and services Adoption of digital technology
Staff/capacity building	Efficiency Performance Sustainability of businesses
Economic	Wealth creation Business revenue Attracting research and development investment
Policy	Public services Policy-making Legislation

Using these impact types allowed us to reveal the so-called non-academic impact, both positive and negative, of academic research in three nature conservation organisations (Table 2). Non-academic impact is defined here as a *demonstrable effect on or change* to society, professional practice, staff/capacity building, economy and policy *beyond academia*. While the various research councils—whose documentation we used to identify the above-named key areas—typically aim to capture impact to demonstrate the widest possible benefits of research conducted, we used this initial framework to determine both positive and negative impacts of working with academia on partner organisations. We used interviews with key informants as a means through which the partnering organisations could provide the evidence for each of the impacts that the collaboration may have had; interviewing took place at an advanced stage of the projects, after two (SMI) to four (RSPB, BBCT) years of partnership working.

**Table 2:** Summary of positive and negative impacts extracted from interviews with staff in three nature conservation organisations which collaborated with academia

	Positive impacts	Negative impacts
Working relationship	Breath of expertise in a small but coherent team Exposure to new disciplines Quick and constant progress Fluid and flexible approach to project management Individual and organisational learning Adoption of new expertise	By times difficult to keep abreast of development Lack of clarity on expectations and obligations Difficulty of pleasing different organisational objectives Time (and resources) required to learn new skills for adopting new technology
Social	Improved monitoring of volunteer engagement and retention Improved support for volunteer training	Created animosity among volunteers due to changes in reporting practices (including the need to use an online platform)
Professional practice	Adoption of innovative digital technologies More accurate information Streamlined workflows and practices	Relocation of workload onto different areas that may require re-skilling of staff
Staff/capacity building	Development of new skills Release staff from time-consuming duties Realising different ways to carry out business Reallocation of freed up time to other pressing issues	Learning new technologies can be time intensive
Economic	Efficiency-savings through improved workflows Income generation Staff time savings	Ongoing support for new technology, both in terms of its cost and required expertise
Policy	Too early to tell, so far it allowed organisations to systematically gather data that could influence future policy	None reported
Emergent impacts	Increased data processing capabilities Increased awareness of pressures that academia is under Realisation of possibilities and limitations of IT infrastructures	Created the need to assign more resources to data processing-dependent activities A realisation for partners that academia is heavily influenced by funding opportunities Increased layer of complexity in managing conservation objectives as a result of an additional partner

### Interview approach

Our data were gathered through in-depth semi-structured interviews with nine staff across the three partner organisations. Staff were selected by sourcing all relevant partners’ details from each of the projects and discussing each contact’s role in, or relevance to, the project. Those interviewed were staff central to the collaboration (i.e. holding a clear ‘stake’—Mitchell et al. 1997), and in all cases these staff had a role in the executive team (ET) or the management team (MT) of their respective organisations, which meant that they could directly affect the direction and scope of the collaborations with academia. Once identified, the staff were approached by an independent researcher, dot.rural’s Impact Research Fellow, who was not part of any of the existing project teams.^1^

### Interview structure and content

All interviewees were first approached via email/telephone to schedule a convenient time and location for an interview; none of those contacted declined the invitation to be interviewed. At the start of the interview, all participants were given an ‘informed consent form’ that described the purposes of the interview and asked for their voluntary participation, their permission to record the interview and that they granted their responses to dot.rural for research purposes in anonymity. All the participants agreed to the conditions and signed the consent form.

Once informed consent was obtained, the interview started by exploring the nature of their working relationship with the respective dot.rural team (first section) and the impact, both positive and negative, that the partnership working may have had on their organisations (second section). In the first section, interviewees were actively prompted to elaborate on: the challenges encountered to get to the desired objectives; differences between this collaboration and working with other practitioners; any learning as a result of the collaboration; and any reservations about the collaboration. The second section dealt with the non-academic impact that the collaboration may have had on the organisation in terms of their societal engagement, professional practice, staff/capacity building, economy and policy (with impact hereafter viewed as change in one or more of these dimensions). In the final phase of the interview, interviewees were given a further opportunity to complement their responses and ask questions. The interviews lasted on average 45 min.

### Data analysis

A professional transcriber transcribed the interviews verbatim; these transcripts were checked for accuracy and subsequently imported into MAXQDA 11 (MAXQDA, 1984–2014), a computer-assisted qualitative data analysis software program. We followed the recommendations of Kelle (2007) who suggests that qualitative data analysis should include both pre-defined and emerging coding. The pre-defined coding followed the two-section structure of the interviews: challenges, reservations, differences and learning for the first section; and positive and negative impacts from working with academics for the second section (see Table 1). *Emergent* coding was created through repeated interaction between the coder and the data. This allowed us to break the “data apart and delineating concepts to stand for blocks of raw data” (Corbin and Strauss 2008, p. 195) that were not considered a priori in the interview questions.

## Results

### Section 1: Working relationships

#### Reservations, differences and challenges

Our partners indicated that the main difference of working with academics, as opposed to their usual working relations, was the academics’ ability to bring a breadth of expertise into a coherent team: “*I was always very impressed with the sort of collaborative set up that they had, that they were able to bring all these disciplines together in quite a small team* […] *it certainly changed my outlook* […] *I had not dealt with other bodies or organisations that brought that width or breadth of outlooks*” [RSPB, MT]. One of the main differences of these collaborations was that it brought the partner organisations in close contact with academic disciplines new to them: “*the simplest and most obvious way it differs, I guess is* […] *that it* […] *involves* […] *working with disciplines, particularly in the computer sciences, that we would never normally work with. I mean that’s* […] *the single biggest difference, I think*” [RSPB, ET].

The academics were seen as generally more independent than the organisations’ usual working partners: “*dot.rural are more self-sufficient in a way than a lot of other partners we had to do a lot of involvement with* […] *we had to collaborate quite a lot but once set up the dot.rural* [team] *has been able to keep it going almost independently from us.*” [BBCT, MT]. Although co-working was perceived to be at the centre of the work by interviewees, the independent working style of academics meant that progress was ongoing: “*the tool evolves without us saying* […] *because there’s constantly improvements being made*” [BBCT, ET]. The downside of this was that instead of investing time managing the work, partners had to invest in catching-up: “*things can move on at a fast pace so sometimes you’ve got to spend a bit of time just trying to keep up with how people are developing things*” [SMI, ET].

A recurrent reservation expressed about working with academics related to the more ad-hoc nature of the collaboration compared with their regular working partners: “*…with a change of staff, if there hadn’t been a handover period when our Chief Exec left us we* [wouldn’t have been] *quite sure what the history of the relationship had been* [including] *who are these Aberdeen folk and what is it they’re doing for us and why have we not written down what we expect to get and what they expect to get?*” [BBCT, ET]. This situation repeated itself with further personnel changes and prompted the organisation to make explicit the working arrangements and objectives of the collaboration to ensure the sustainability of the project: “*So I’ve come into a project part-way through without the required history. So that was the major challenge for me* […] *to start from the beginning and to understand* […] *what has happened, what was the priorities for the project, where was it going. So I didn’t have that information. So that was* […] *the key challenge for me*” [BBCT, ET].

Interviewees mentioned that one further challenge of this type of collaboration was the marrying of different objectives: academic and organisational. Ultimately these all seemed to relate to communication within the team: “*Communication can be a challenge, its mainly communication between what the practical manager wants, a practitioner wants and what does an academic want* […] *there were certain times when there were strains between staff, practitioners and dot.rural staff about how the database was developed and how it looked on the screen* […] *so that’s sometimes where I had to kind of intervene a little bit to sit and talk to actually try and find out what are the ways of being able to solve this, what was the actual nature of the problem*” [SMI, ET].

#### Learning

Interviewees brought out different learning outcomes as a result of the collaboration, ranging from the individual: “*I finally realised the potential of the internet and* […] *crowd-sourcing in particular in solving problems to do with ecology*” [BBCT, MT]; *to the organisational: “from an organisational point of view we’ve discovered with the help of dot.rural because they’ve really, really, really helped us develop it to the point where I think we’ve got a* […] *kind of unique system in that we can produce the kind of reports that we’re producing, we can also feedback to the volunteers as well*” [SMI, MT]. Furthermore, in some cases the collaborations pushed the partner organisations into adopting new areas of expertise: “*I’d never come across phpMyAdmin before.* […] *So therefore I’ve never had a strong desire to learn how to use it* […] *But I know for a fact that once I can do what I need to be able to do I will enjoy it, because I’ll know* […] *I’ll feel more in control and less dependent on other people*” [SMI, MT].

The collaboration with academics also gave partners a better understanding of applications from the research world: “[…] *the sort of initial results that have come out of the research that’s been happening as to how much feedback you give to people, and how that affects whether they repeat use, is really useful for all of our communications* […] *if we don’t give enough or we give too much information that can affect our blogs, our E-newsletters, what we put in our members magazine, it’s not just how much you feedback through the BeeWatch tool.* […] *it’s useful feedback for any communication with people regarding citizen science or nature conservation generally.*” [BBCT, ET]; and “*I’ve learnt a lot about what is possible to do and what could be possible to do and I’m quite excited about that.* […] *I do think it’s the way to go in terms of* […] *getting more effective and more efficient management systems is to get these closer links with the research*” [SMI, ET].

### Section 2: The organisational impacts of collaborating with academia

#### Social impacts

In general, the identified social impacts discussed by the interviewees revolved around volunteer engagement and citizen science. Interviewees from both SMI and BBCT indicated that the systems created through the collaborations allowed them to monitor volunteer engagement and retention: “*this system ties* […] *identification of priority areas where we can actually say* […] *if volunteers are going to start getting bored and dropping off then we can start looking at which of the areas we really need to put an effort into [to] make sure they stay on-board and stay monitoring those areas.*” [SMI, MT]. For the BBCT, the system developed was a multi-faceted tool to be used to train novices as well as the more experienced users in a supportive environment: “*the tool’s a great sort of first step into it, if you get somebody who’s enthusiastic about bumblebees but doesn’t feel confident enough to go out and walk a transect once a month you can introduce them to it by saying: right well if you’re out on a walk take a picture, go on use the online training tool. Then once you feel a bit more confident* […] *sign up and do a transect once a month, you can still be using the tool to identify the specimen that you see on your walk so if you see something and you’re not sure take a picture, use the tool to identify it and put that on your records*” [BBCT, ET].

Similarly, the RSPB highlighted the citizen science impacts: “*I expect that the people who looked at it and I know when we put out the publicity* […] *a lot of folk came back and said oh this is brilliant! This is really interesting so perhaps the same* […] *light bulb that went off in my mind went off in lots of other people’s minds as well and they thought oh that’s quite good* […] *with something like this I think it’s very well set up to go to schools with and you could talk to them about the kites and you can talk to them about satellite tracking, but you can talk to them about technology as well and yeah* […] *all those people will know a lot more about technology than I do!*” [RSPB, MT].

However, for SMI, the digital solution generated tension with some of their volunteers who did not wish to move onto the digital platform: “*a lot of our volunteers are ghillies, gamekeepers, that sort of people which* […] *tend to be fairly conservative and resistant to change. So you know they’re typical… ‘I’m not going to report things on line; I like talking to people!’*” [SMI, ET].

#### Professional practice impacts

All collaborations led to the adoption of digital technologies and innovation in products and services, as systems were created that streamlined the processes around data workflows: submission, handling and archiving. The new data workflows allowed one partner organisation to have, for example, “*a better understanding of the distribution of the bees because one thing we really need to understand is just exactly where the bees are*” [BBCT, MT]. The streamlining of data submission meant that there was less space for error because the data went into the database without staff intervention: “*instead of manually do all the stuff and put it in Excel spreadsheets and so on, the dot.rural team set it up so it does it all automatically, so the data comes in and its mapped*” [RSPB, MT]; and “*it’s fundamentally changed how we operate* […] *because its provided an online resource that instead of people reporting directly to us and us compiling the information they report directly to the database and we compile it from the database, which is a lot easier and a lot more efficient for us to do*” [SMI, ET].

Another aspect of professional practice was that these new systems helped reshape organisational priorities by providing them with the means for new ways of operating: “*We’ve been changing to become more scientific* […] *in general—more of a data provider than just doing conservation management* […] [the new platform] *and the data that we get out of that is a major part of that, alongside data that we get from other sources*” [BBCT, MT].

One downside of the new workflows for SMI and BBCT was that it created dependency on the new system by shaping the workload priorities of staff, for example: “*in the height of the summer we do have to bring other staff in* […] *so our outreach officer had to feed into doing some of the IDs over the summer just because there were so many records and we were falling behind and we didn’t want there to be a delay between people uploading and getting the feedback.* […] *But you know the data we get from it and the engagement aspect of it it’s worth it. Um…its…you know it’s part of…we just have to plan that in that that’s part of our workload now*” [BBCT, ET]; and “*reporting is my least favorite thing because…of the process, you know, I’ve got to tidy it up before I can use it. Well, I dunno, maybe that’s normal, but, ehm…it just slows everything down. But also, I need to…you know, I need to improve my skills so as I can use it better, because there are queries that we could run, that would save me…counting stuff in Excel*” [SMI, ET].

#### Staff/capacity building impacts

The collaboration with academics generated impacts related to efficiency, performance and organisational sustainability, and was seen to benefit the organisations by delivering expertise that helped them save time, get more accurate data and release staff from duties that now could be achieved through automated systems: “*it’s made a difference just in terms of other things that I’m able to do, so instead of spending time on constantly answering queries I can be answering questions about other things or delivering events*” [BBCT, MT]. Indeed, the freeing up of direct and additional staff time was a recurrent perceived benefit flowing from the collaborations: “*a member of dot.rural will be logging in and identifying species one day per week which is great, so that’s probably one of the few projects where we’ve got somebody from an external organisation logging in and helping us do our work*” (BBCT, ET); and “*they were doing us a service because they were dealing with all this stuff that we didn’t have time to deal with*” (RSPB, MT).

The collaborations appeared to have helped the organisations realise avenues for future work: “*There’s been a realisation that there’s a lot of potential there, which the RSPB simply doesn’t explore and doesn’t understand how to make the best of at the moment. I think that… in terms of mind set and* […] *opening of eyes you could say there has been an impact.*” [RSPB, ET]. Similarly, they resulted in noticeable changes to individual staff members in terms of personal development and skill-sets, but this came at the cost of having to invest in re-skilling and training staff. For example: “*looking at things from a research point of view I see the need now to think of clear questions that you need answered and guiding how you produce the database and things like that* […] *to answer the questions that you need to be answered so I think that’s really important. I mean personally I’ve learnt so much about database management, data management, tidying data and that sort of thing and that’s invaluable to me*” [SMI, MT]; and “*I’ve kind of finally realised the potential of the internet and crowd sourcing, in particular in solving problems to do with ecology* […] *it’s just been a real introduction to ways in which those can be fixed*” [BBCT, MT].

#### Economic impacts

The interviewees described economic impacts in terms of efficiency-savings, income generation and staff time. For example: “*because of the automated system it means we can answer queries quite quickly* […] *from maybe three or four minutes down to about 30 seconds*” [BBCT, MT]; and “*data entry used to be* […] *somewhere around 25 % or more of their time* […] *and data analysis was just a nightmare.* […] *Using the data we can draw out of it now* […] *we’re down to less than 10 % of our time.*” [SMI, ET]. SMI required the newly developed infrastructure to be used by all its partner organisations involved in mink control across large parts of Scotland, which generated efficiency-savings on a range of fronts (data entry, archival and analysis, reporting): “*all trusts that submit data ought to use the system as a condition for payment*” [SMI, ET]. Yet, for the RSPB, “*the collaboration is* […] *probably* […] *you know, too small-scale in a large organisation for it to be measureable at that* [economic impact] *level*” [RSPB, ET].

Where occurring, the economic impacts did not necessarily stop within the boundaries of the organisations; for the BBCT the collaboration with academics may have given them the competitive edge to secure further funding: “*we have a three-year grant from the Esmèe Fairbairn Foundation for a project and we mentioned in our funding application that we have this tool we developed and what it does. Part of the role of the person funded by this would be to manage that tool and help expand it* […] *so whether they thought that was a beneficial aspect of the project and that was part of the reason they gave us the three years of funding I don’t know*” [BBCT, ET].

Despite the emergence of general ‘cost savings’, there was also recognition that the collaborations had a significant cost “*it’s a hell of a lot of work especially if you don’t have anyone in the project that is that kind of…computer savvy or doesn’t have the time available or something like that*” [SMI, MT] and that once the collaborations ended, the organisations would have to absorb the cost of running the new technologies: “*to the detriment, in the future, I think it might!! [Laughs] ‘Cause we’re going to have to* […] *resource things that* […] *now they’re coming to the end of dot.rural, that we* […] *we hadn’t anticipated*” [BBCT, ET].

#### Policy impacts

The interviewees generally felt that policy impacts had not yet been realised: “*Simply too early to expect to be able to see those sorts of impacts*” [RSPB, ET]; and “*we should have to be careful on what sort of timeframes we look at to get these quite* […] *large impacts because if you’re looking for an impact on policy it can take you years to get that*” [SMI, ET]. At the same time, however, the interviewees indicated that it would only be a matter of time before their organisations would start influencing policy through the new workflows. All of the collaborations were building up a data corpus in order to influence policy in the longer term; for example: “*the idea is that it [the work with dot.rural] will feed into the government recording schemes* [such as the] *National Pollination Strategy which DEFRA are working on at the moment* […]. *We’re involved with some of the outcomes from that and data provision. Because essentially, that’s the only way that we’ll know* […] *what bee species are where—we’ve not got enough data on them at the moment* […] *and this is a way of filling in those gaps*” [BBCT, MT].

### Emerging impacts

The second phase of data analysis brought out three further *emergent* nodes outside the focus of our impact framework, namely *awareness raising*, *expectation management* and *data accuracy*.

#### Awareness raising

The collaboration helped the partners understand the possibilities and limitations of IT infrastructures: “*some of the* […] *staff on our side were a little bit naive in terms of how simple people think it is to set up an online database* […] *any database actually because everybody thinks ‘oh digital stuff, no problem, database’, you just do the database and it will work, nah it never works like that!*” [SMI, ET]. It also taught the interviewees about practicalities of their own organisations’ culture: “*you just hit the glue sometimes* […] *it was very easy those first few years because it was me and dot.rural in Aberdeen and we would just email or phone and say can we meet up next week and we would do it. But moving into this next phase I cannot do that myself,* […] *we don’t have the authority to go and do that just in this office*” [RSPB, MT].

#### Expectation management

Through working together the teams had a window into each other’s daily demands, thus allowing them to set manageable expectations: “*I think there’s* [now] *a lot better understanding between the two different groups* [academics and practitioners] *of what the pressures on each of them are. I think that’s the main thing and we certainly have a better understanding of the pressures the dot.rural group are under*” [SMI, ET]; and “*with academia, that things can move away from their original focus because* […] *other ways have come up or more research* […] *more viable research has come up. So, it’s more like shifting sand*” [BBCT, ET].

#### Data accuracy

The infrastructures developed with the partners enhanced their organisation’s confidence in the accuracy of (biological) records generated and therefore their value for nature conservation: “*the problem with this species is that we don’t know very much about their distributions* […] *because the country is so big and because so few people are really good at identifying them… we want to be able to identify them ourselves but not have to go out in the field and look for them everywhere so this really just allows us to effectively cover the whole country in terms of surveying because we can see where things are*” [BBCT, MT]; and “*it’s definitely increased the amount of records that we’re getting in so we get better from an overall project management point of view. We can clearly see now from looking at the data that some areas are clearing* [from American mink], *some areas are increasing and some there haven’t been any catches.* […] *so that’s really improved it because we can clearly see patterns now in the data*” [SMI, MT].

## Discussion

In this paper we have explored and identified from a user perspective the impact, both positive and negative, of developing innovative digital technologies in nature conservation organisations through partnership working with academia. Using a ‘non-academic’ impact methodological framework, we showed how working with academics could translate into tangible social, professional practice, economic, capacity building and policy impacts.

Digital technologies are known to have the potential to improve workflows for data acquisition, data management and data reporting. For us to reveal such positive impacts from collaborations with academia in nature conservation through a qualitative impact evaluation was therefore not surprising and echoes similar findings from within this realm (Dickinson et al. 2012; Miller-Rushing et al. 2012). However, the collaborations with academia we investigated generated a range of impacts for partner organisations that went well beyond those commonly reported efficiencies that may come with innovative digital technologies, and concerned both positive and negative aspects.

Adopting innovative digital technologies can affect professional practice (Bonney et al. 2009; Arts et al. 2013); in our case studies, we found that these have potential economic benefits via reductions in operational costs while at the same time building capacity in the organisations through the generation of new knowledge and skills, reallocation of resources and maximising the use of available data. We also found that for these organisations the positive impacts can help them engage more efficiently with their current, as well as new, audiences through novel feedback mechanisms that automate the previous time-consuming processes (see for example Blake et al. 2012; Newman et al. 2012).

Critically, the disciplinary mixture of the academic teams and inclusion of computing science therein appeared to have brought the collaborating practitioners to different places, from which they could see a world of new opportunities. The virtues of partnership working have been sung widely, particularly in the context of so-called transdisciplinary research (e.g. Lawrence and Despres 2004; Pohl 2005; Wickson et al. 2006). This extensive body of literature has made it clear that practitioners have a lot to offer to the process of knowledge acquisition (e.g. Irvine et al. 2009; Phillipson et al. 2012), notably widening the horizon of otherwise perhaps too focused academics. While the latter was outside the scope of our investigation, our research does provide evidence for its mirror image, with academics from different disciplines changing perspectives of practitioners, in our case in the nature conservation realm.

Co-creating digital solutions with academics to enhance the delivery of conservation objectives clearly saddled partner organisations with an investment debt. Hence, what was identified as a positive influence on professional practice at the time (with the academics still around to support the digital technology) may ultimately force investment by an organisation to maintain new forms of working when the collaboration has come to an end. This may mean a fundamental change in the type of personnel appointed in nature conservation organisations. For example, in organisations with few operational staff this may even lead to appointing more technology-oriented personnel at the expense of the more traditional conservation-oriented staff (Arts et al. 2013).

It is plausible that such path dependencies are more likely to flow from collaboration with academia than from purchased digital technologies (e.g. through consultancy agreements with an IT firm), although the latter is also known to lead to changes in staff skill profiles (Kamal 2006). Rather than having asked for a specific digital solution to a known problem, the co-working with academia brought practitioners to new places and may have drawn them to more sophisticated services, and thereby made radically new working practices disproportionally attractive. Again, for small organisations, personal investment in the collaboration with academia may mean that the adoption of innovative digital technology has allowed the organisation to move on without there being a way back (Wolcott et al. 2008). Whether this represents a dilemma or a technological imperative that the organisation positively embraces, financially plans for and invests in remains a contentious issue. For example, the BBCT now receives in excess of 800 records per month for verification and, although this gives them the desired database to start influencing policy, it requires them to reallocate resources to match the new demand. Similarly, for SMI the developed digital solution has become an integral part of their operations to the point that it is a condition for payment for some of their partners.

Given the diversity of digital technologies that may be used for user engagement (and citizen science), it has become critical that organisations consider the types of technologies that they wish to pursue. There are several examples in the literature with regard to the risk of public engagement fatigue with digital technologies (Newman et al. 2012; Roy et al. 2012). Our interviewees mentioned that although this can be the case, when looked at from a management perspective it is paradoxically the same technology that can be used to reinvigorate engagement by refocusing volunteer engagement efforts onto those areas where volunteers’ data show little or no activity over significant periods of time.

A recurrent message flowing from our interviewees to those organisations wishing to embark upon collaborations with academia was that sufficient time should be put aside at the beginning of the collaborations for interaction (face-to-face and otherwise) to appreciate differences in working practices and goals, as well as to get most out of the shared journey ahead. This resonates with the work of Moon and Blackman (2014) who, in advocating that different disciplines need to share ontological, epistemological and philosophical orientations for successful integration, eloquently describe the basis of not only interdisciplinary but also transdisciplinary work. It is this early part of the collaborative process, we argue, during which the seed of impact is sowed by allowing partners the space to share and mould different perspectives into a shared vision.

## Conclusion

Collaborations with academia have allowed nature conservation organisations access to new digital technologies to help deliver their conservation objectives. Using in-depth interviews with staff from nature conservation organisations we revealed that, through working with academics, conservation organisations could receive positive and negative impacts. Positive impacts such as new ways of engaging with audiences, improved data workflows, capacity building and the development of digital infrastructure to help them influence policy and obtain financial benefits were accompanied by negative impacts in terms of the time and resources required to learn new skills and sustain new technologies, managing different organisational objectives and the need to shift working practices as a result of the new technologies. Most importantly, however, collaboration with academia situated practitioners in multidisciplinary environments, bringing them to different places from which they could see a world of new opportunities with regard to the application of novel technologies within their organisations and beyond.

## Footnotes

1 The role of the Impact Research Fellow was to ensure that the non-academic impact of dot.rural was documented and fed back to RCUK, while also critically capturing the wider issues around the digital innovation work conducted by dot.rural.
